# Phase II study of FOLFIRI with low-dose irinotecan plus ramucirumab as second-line treatment in Japanese patients with metastatic colorectal cancer (study rindo)

**DOI:** 10.1093/jjco/hyaf198

**Published:** 2025-12-08

**Authors:** Norifumi Hattori, Goro Nakayama, Shinichi Umeda, Takayoshi Kishida, Shigeomi Takeda, Kazuhiro Ezaka, Masayuki Tsutsuyama, Mitsuru Sakai, Masashi Hattori, Takeshi Ito, Mitsuro Kanda, Chie Tanaka, Kenta Murotani, Masahiko Ando, Yasuhiro Kodera

**Affiliations:** Department of Gastroenterological Surgery, Nagoya University Graduate School of Medicine, 65 Tsurumai-cho, Showa-ku, Nagoya, Aichi, Japan; Department of Gastroenterological Surgery, Nagoya University Graduate School of Medicine, 65 Tsurumai-cho, Showa-ku, Nagoya, Aichi, Japan; Department of Gastroenterological Surgery, Nagoya University Graduate School of Medicine, 65 Tsurumai-cho, Showa-ku, Nagoya, Aichi, Japan; Department of Gastroenterological Surgery, Nagoya University Graduate School of Medicine, 65 Tsurumai-cho, Showa-ku, Nagoya, Aichi, Japan; Department of Surgery, Tosei General Hospital, 160 Nishioiwake-cho, Seto, Aichi, Japan; Department of Surgery, Yokkaichi Municipal Hospital, 2-37 Shibata 2-chome, Yokkaichi, Mie, Japan; Department of Surgery, Komaki City Hospital, 1-20 Jobushi, Komaki, Aichi, Japan; Department of Surgery, Ichinomiya Municipal Hospital, 2-2-22 Bunkyo, Ichinomiya, Aichi, Japan; Department of Surgery, National Hospital Organization Nagoya Medical Center, 4-1-1 Sannomaru, Naka-ku, Nagoya, Aichi, Japan; Department of Surgery, National Hospital Organization Toyohashi Medical Center, 50 Hamamichikami, Iimura-cho, Toyohashi, Aichi, Japan; Department of Gastroenterological Surgery, Nagoya University Graduate School of Medicine, 65 Tsurumai-cho, Showa-ku, Nagoya, Aichi, Japan; Department of Gastroenterological Surgery, Nagoya University Graduate School of Medicine, 65 Tsurumai-cho, Showa-ku, Nagoya, Aichi, Japan; Biostatistics Center, Kurume University, 67 Asahimachi, Kurume, Fukuoka, Japan; Center for Advanced Medicine and Clinical Research, Nagoya University Hospital, 65 Tsurumai-cho, Showaku, Nagoya, Aichi, Japan; Department of Surgery, National Hospital Organization Nagoya Medical Center, 4-1-1 Sannomaru, Naka-ku, Nagoya, Aichi, Japan

**Keywords:** metastatic colorectal cancer, ramucirumab, FOLFIRI, Japanese patients, second-line chemotherapy

## Abstract

**Objective:**

This multicenter, single-arm, Phase II study aimed to evaluate the efficacy and safety of fluorouracil, levofolinate, and irinotecan (150 mg/m^2^, standard dose in Japan) (FOLFIRI) plus ramucirumab (RAM) as second-line treatment for metastatic colorectal cancer (mCRC) in Japanese patients.

**Methods:**

On Day 1 of each 2-week cycle, patients with unresectable mCRC who were refractory to oxaliplatin and fluoropyrimidine in combination with bevacizumab or anti-epidermal growth factor receptor antibodies as first-line treatment received 8 mg/kg RAM, followed by the FOLFIRI regimen with low-dose irinotecan (150 mg/m^2^). The primary endpoint was progression-free survival (PFS), and secondary endpoints were overall survival (OS), treatment compliance, and safety.

**Results:**

A total of 62 patients were enrolled from 15 institutions between January 2018 and August 2021. The intent-to-treat and safety populations included 61 and 58 patients. Median PFS and OS were 5.9 months (95% CI, 4.8–6.9 months) and 17.0 months (95% CI, 12.0–21.0 months), respectively. The objective response rate and disease control rate were 8.2% and 74%, respectively. The median time to treatment failure was 4.8 months (95% CI, 3.2–5.9 months). The median relative dose intensities of irinotecan, 5-fluorouracil, and RAM were 73.8% (range, 40.3%–102.4%), 58.5% (range, 22.8%–102.4%), and 80.8% (range, 36.1%–102.4%), respectively. Frequencies of Grade ≥ 3 hematologic, non-hematologic, and RAM-associated adverse events were 43%, 24%, and 17%, respectively. The observed Grade ≥ 3 adverse events included neutropenia (40%), diarrhea (8.6%), decreased appetite (10%), hypertension (6.9%), and proteinuria (3.4%).

**Conclusion:**

FOLFIRI with low-dose irinotecan plus RAM is a feasible second-line treatment in Japanese patients with mCRC.

## Teaser

FOLFIRI plus RAM therapy performed in the setting of irinotecan 150 mg/m^2^ can be safely administered without decreasing the therapeutic effect and achieve good survival.

## Introduction

Colorectal cancer (CRC) is the third most common cancer, with an incidence rate of 10%. CRC is associated with a mortality rate of 9.4% worldwide, which makes it the second most common cause of cancer deaths following lung cancer [1]. The standard treatment for unresectable metastatic colorectal cancer (mCRC) is systemic chemotherapy, and the efficacy of combination therapy with molecular-targeted agents as first-line treatment has been reported for bevacizumab (BEV), cetuximab, and panitumumab [2]. Several Phase III trials of second-line combination therapy with molecular-targeted agents after first-line treatment with BEV demonstrated significant improvements in overall survival (OS) [3–5]. Based on these results, combination therapies with angiogenesis inhibitors such as BEV, aflibercept, and ramucirumab (RAM) have become standard second-line treatments [2].

RAM is a fully human immunoglobulin G1 monoclonal antibody that binds to vascular endothelial growth factor (VEGF) receptor-2, preventing VEGF-A, VEGF-C, and VEGF-D ligand binding and inhibiting tumor angiogenesis by suppressing endothelial cell proliferation, migration, and survival [6]. The RAISE trial demonstrated a median OS of 13.3 months in patients treated with fluorouracil, levofolinate, and irinotecan (FOLFIRI) plus RAM versus 11.7 months in those treated with FOLFIRI plus placebo [hazard ratio (HR), 0.844; 95% confidence interval (CI), 0.73–0.98; log-rank *P* = .02] [5]. The irinotecan dose used in the FOLFIRI regimen was 180 mg/m^2^, which is higher than the recommended dose of 150 mg/m^2^ in Japan [7–9]. In the RAISE trial, median relative dose intensities (RDIs) of RAM and irinotecan were 88.2% and 77.0%, respectively, in all patients, and 82.9% and 63.8%, respectively, in the Japanese population [10], showing no difference for RAM but a lower RDI for irinotecan in the Japanese population. Moreover, incidence rates of adverse events leading to discontinuation of cytotoxic agents were 26.8% in all patients and 48.6% in the Japanese population [10]. Based on these results, we conducted a prospective trial to investigate the efficacy and safety of FOLFIRI with low-dose irinotecan (150 mg/m^2^, standard dose in Japan) plus RAM as second-line treatment for mCRC in Japanese patients, including those previously treated with BEV or anti-epidermal growth factor receptor (EGFR) antibodies. This study aimed to determine whether this lower irinotecan dose could maintain comparable efficacy while improving tolerability.

## Patients and methods

### Study design

This multicenter, single-arm, Phase II clinical trial was conducted by the Chubu Clinical Oncology Group (CCOG) at 15 hospitals in Japan. Inclusion criteria were as follows: age ≥ 20 years; histologically proven adenocarcinoma of the colon or rectum; unresectable metastasis; confirmed radiographic disease progression during first-line combination therapy of oxaliplatin (OX) and fluoropyrimidine with BEV or anti-EGFR antibodies; the presence of at least one measurable lesion according to the Response Evaluation Criteria in Solid Tumors (RECIST version 1.1); Eastern Cooperative Oncology Group (ECOG) performance status of 0 or 1; and adequate hematologic, hepatic, and renal function. Patients who had received first-line treatment with IRI and had uridine diphosphate glucuronosyltransferase *(UGT)1A1* gene status of homotype (^*^28/^*^28, ^*^6/^*^6) or double hetero-type genetic polymorphisms (^*^6/^*^28), brain metastasis, clinically significant cardiovascular disease, double cancer, bowel obstruction, uncontrolled diabetes mellitus, hypertension, or congestive heart failure were excluded.

This study was conducted in accordance with the principles of the Declaration of Helsinki. All patients provided written informed consent prior to participation. The ethics committees of Nagoya University Hospital (approval number 2016-0527) and all other participating facilities approved the study. This trial was registered with the University Hospital Medical Information Network (UMIN000025659) and Japan Registry of Clinical Trials (jRCTs041180074).

### Treatment plan

On Day 1 of each 2-week cycle, patients with unresectable mCRC after first-line combination therapy of OX and fluoropyrimidine with BEV or anti-EGFR antibodies received 8 mg/kg RAM as a 60-min intravenous infusion, followed by the FOLFIRI regimen with low-dose irinotecan (150 mg/m^2^ intravenous irinotecan given over 90 min, 200 mg/m^2^ intravenous leucovorin given over 120 min, 400 mg/m^2^ fluorouracil given as an intravenous bolus over 2–4 min, and 2400 mg/m^2^ fluorouracil given as a continuous infusion over 46 h). The treatment was continued until any one of the following occurred: radiographic confirmation of disease progression, unacceptable toxicity, deterioration of ECOG performance status to > 2, or withdrawal of patient consent. Dose modifications reflecting treatment-related toxicities were carried out in accordance with the study protocol.

### Assessments

The primary endpoint was progression-free survival (PFS), defined as the time from the date of registration until disease progression or death from any cause. Secondary endpoints were OS, defined as the time from the date of registration until death from any cause; time to treatment failure (TTF), defined as the time from the date of registration until death from any cause or cessation of the protocol treatment; objective response rate, defined as the proportion of patients whose best response was complete response (CR) or partial response (PR); disease control rate (DCR), defined as the proportion of patients whose response was CR, PR, or stable disease (SD); treatment compliance; and adverse events.

Tumor size was assessed using computed tomography at baseline and every 8 weeks thereafter. Treatment response was assessed according to the RECIST criteria, version 1.1, by the investigators. Adverse events were graded according to the National Cancer Institute Common Terminology Criteria for Adverse Events, version 4.0. All analyses of efficacy were based on the intent-to-treat (ITT) population, defined as eligible and assessable enrolled patients. The safety population was defined as all patients receiving ≥ 1 dose of the protocol treatment.

### Statistical analysis

Statistical power was calculated based on the following assumptions: a PFS threshold of 4.3 months and expected PFS of 5.7 months based on data from a Japanese subgroup of the RAISE trial [5], with an enrollment period of 2 years and follow-up period of 3 years. To ensure an alpha level of 0.1 (one-side) and a detection power (1 − β) of 80%, 59 patients were required. Therefore, the planned sample size was 60 patients to account for possible loss to follow-up. Time-to-event variables, PFS, OS, and TTF were analyzed by the Kaplan–Meier method. If the lower limit of the 95% CI exceeded 4.3 months, which was set as the threshold PFS, the protocol treatment was considered to be effective. *P* values < .05 were considered statistically significant. Statistical analyses were performed using SAS 9.4 (SAS Institute Inc., Cary NC).

## Results

### Patient characteristics

**Figure 1:**
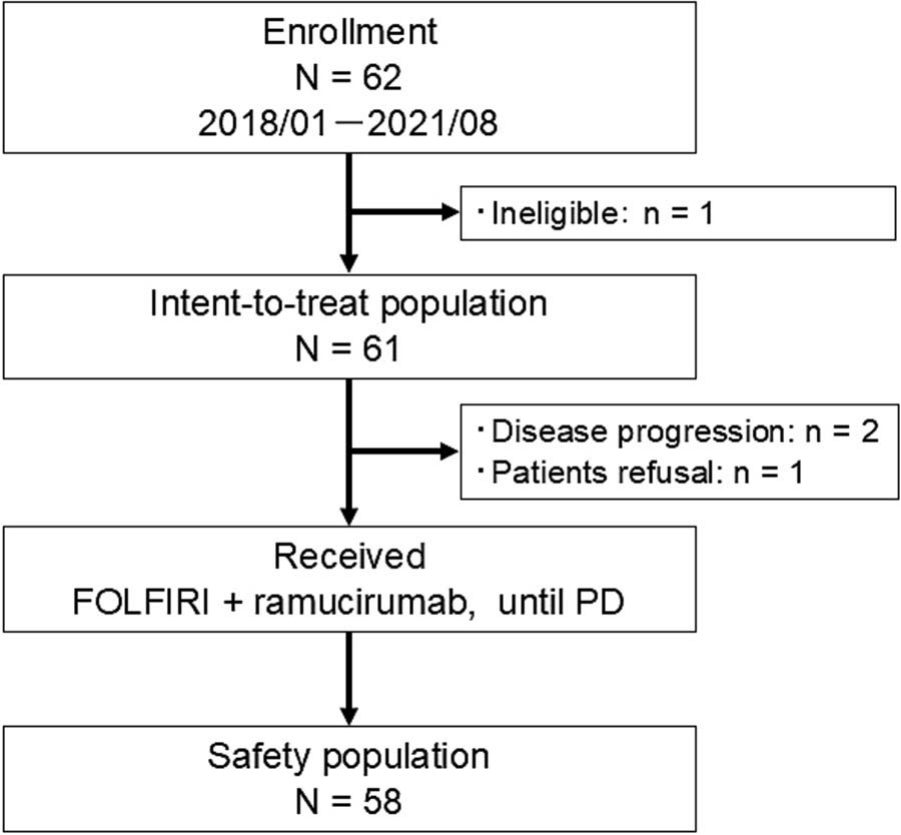
Consort diagram, a total of 62 patients were enrolled between January 2018 and August 2020. FOLFIRI plus RAM chemotherapy was administered to 58 patients. *N*, total number of patients; *n*, number of patients; *FOLFIRI*, fluorouracil, levofolinate, and irinotecan; *PD*, progression disease.

A total of 62 patients were prospectively enrolled from 15 institutions between January 2018 and August 2021. One patient was excluded after enrollment because of ineligibility. The reason for ineligibility was that protocol treatment had been administered before registration. The ITT population comprised 61 patients. Three patients were excluded from the safety analysis, including 2 patients who could not receive chemotherapy because their general condition worsened due to acute disease progression after enrollment, and one patient who refused to receive protocol treatment. Thus, the safety population comprised 58 patients (Fig. 1). Baseline clinical characteristics are summarized in Table 1. Median age of patients was 69 years (range, 43–80 years); 32 patients (52%) were male, and 59 patients (97%) had ECOG performance status 0. The primary tumor location was the right side in 15 patients (25%) and the left side in 46 (75%). The RAS status was wild type in 29 patients (48%) and mutant in 32 (52%). Combination molecular-targeted drugs used in first-line chemotherapy were BEV in 44 patients (72%) and anti-EGFR antibodies in 17 (28%). BRAF mutation analysis was conducted in 44 of the 61 patients, and no BRAF-mutated cases were identified. Thirty-five patients (57%) had two or more metastatic sites, with the most common metastatic site being the liver (61%) followed by the lungs (56%).

**Table 1:** Patient characteristics (*N*, total number of patients; *n*, number of patients; *PS*, performance status; *ECOG*, Eastern Cooperative Oncology Group; *BEV*, bevacizumab; *EGFR*, epidermal growth factor receptor; *UGT*, uridine diphosphate glucuronosyltransferase)

Variable	*N* = 61
Age, years	
Median (range)	69 (43–80)
Sex, *n* (%)	
Male	32 (52)
Female	29 (48)
PS (ECOG), *n* (%)	
0	59 (97)
1	2 (3)
Primary tumor location, *n* (%)	
Right	15 (25)
Left	46 (75)
Primary tumor resection, *n* (%)	
Yes	39 (64)
No	22 (36)
*RAS* status, *n* (%)	
Wild type	29 (48)
Mutant	32 (52)
Previous therapy, *n* (%)	
BEV	44 (72)
Anti-EGFR antibody	17 (28)
Number of metastatic sites, *n* (%)	
1	26 (43)
2	28 (46)
3	7 (11)
Metastatic site, *n* (%)	
Liver	37 (61)
Lung	34 (56)
Distant lymph node	13 (21)
Peritoneum	10 (16)
Other	10 (16)
UGT1A1 polymorphism, *n* (%)	
-/-	30 (49)
-/^*^28 or -/^*^6	29 (48)
Unknown	2 (3)

### Treatment exposure

The median time to treatment failure was 4.8 months (95% CI, 3.2–5.9 months). The median number of treatment cycles was eight (range, 1–27). The median RDI was 50.5% (range, 0%–102.4%) for bolus fluorouracil, 74.5% (range, 28.5%–102.4%) for infusion fluorouracil, 73.8% (range, 40.3%–102.4%) for irinotecan, and 80.8% (36.1%–102.4%) for RAM (Table 2). Common reasons for treatment discontinuation were disease progression (67%), adverse events (16%), and patient decision (8%).

### Efficacy

**Figure 2:**
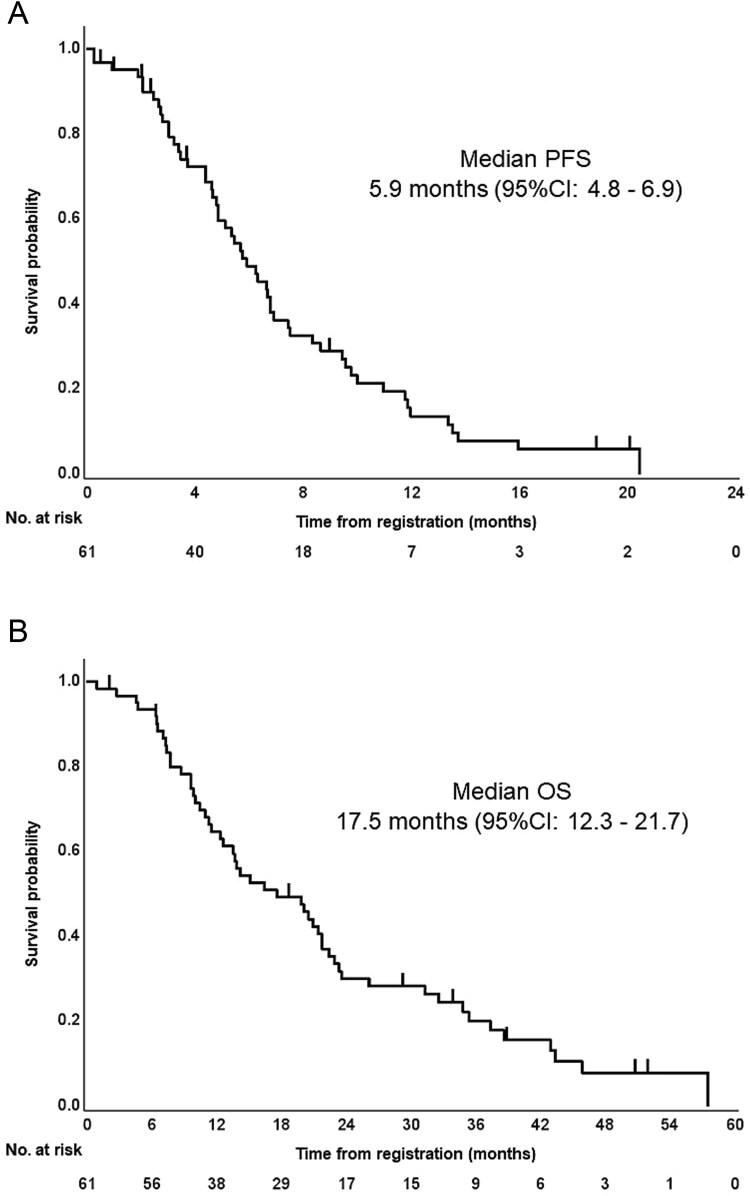
Survival outcomes in the ITT population (*N* = 61). (A) Progression-free survival. Median progression-free survival was 5.9 months (95% CI, 4.8–6.9 months). The lower limit of 95% CI was higher than the threshold of 4.3 months. (B) Overall survival. Median overall survival was 17.5 months (95% CI, 12.3–21.7 months). *PFS*, progression-free survival; *OS*, overall survival; *CI*, confidence interval.

The cutoff date for the primary analysis was December 2023. Disease progression and death occurred in 53 patients (87%) and 52 patients (85%), respectively. The median length of follow-up for censored cases was 16.4 months (range, 1–57 months). At study cutoff, no patients were receiving FOLFIRI plus RAM. Median PFS (primary endpoint) was 5.9 months (95% CI, 4.8–6.9 months) (Fig. 2A). The lower limit of 95% CI was higher than the threshold of 4.3 months, and hence, the primary endpoint was met. Median OS was 17.5 months (95% CI, 12.3–21.7 months) (Fig. 2B). The tumor response was assessed in the ITT population (61 patients): five patients (8.2%) were not evaluated. The CR rate was 0%, the PR rate was 8.2%, the SD rate was 66%, the PD rate was 18%, the objective response (CR + RR) rate was 8.2% (95% CI, 3.6%–17.7%), and the disease control rate (CR + PR + SD) was 74% (95% CI, 61.5%–83.1%).

**Table 2:** Treatment exposure (*N*, total number of patients; *n*, number of patients; *RDI*, relative dose intensity)

	*N* = 58
Treatment cycles, median (range)	8 (1–27)
RDI, %, median (range)	
Bolus fluorouracil	50.5 (0–102.4)
Infusion fluorouracil	74.5 (28.5–102.4)
Irinotecan	73.8 (40.3–102.4)
Ramucirumab	80.8 (36.1–102.4)
Dose modification, *n* (%)	
Irinotecan	
Reduction	30 (51.7)
Delay	48 (82.7)
Ramucirumab	
Reduction	8 (13.7)
Delay	45 (77.5)

**Figure 3:**
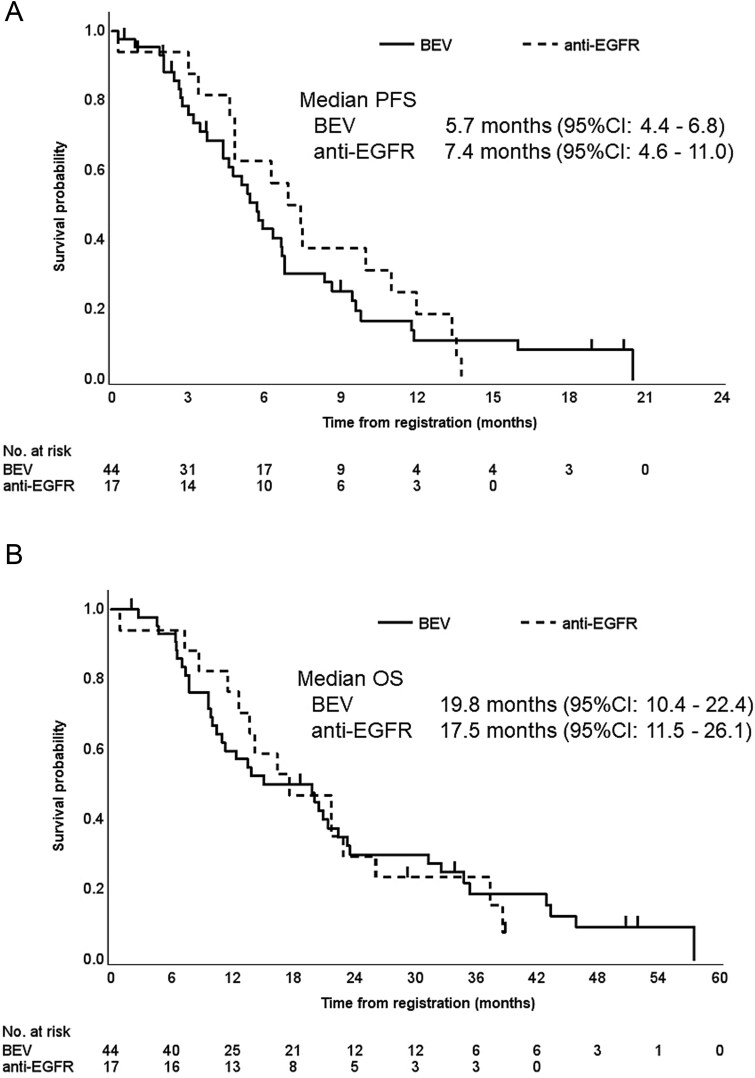
Survival outcomes by previous therapy (BEV or anti-EGFR antibodies). (A) Progression-free survival. Median progression-free survival was 5.7 months (95% CI, 4.4–6.8 months) in patients treated with first-line chemotherapy with BEV and 7.4 months (95% CI, 4.6–11.0 months) in those treated with first-line chemotherapy with anti-EGFR antibodies (HR, 1.17; 95% CI, 0.64–2.12; P = .60). (B) Overall survival. Median overall survival was 19.8 months (95% CI, 10.4–22.4 months) and 17.5 months (95% CI, 11.5–26.1 months), respectively (HR, 0.96; 95% CI, 0.52–1.78; *P* = .91). *PFS*, progression-free survival; *OS*, overall survival; *CI*, confidence interval; *BEV*, bevacizumab; *EGFR*, epidermal growth factor receptor.

Median PFS was 5.7 months (95% CI, 4.4–6.8 months) in patients treated with first-line chemotherapy with BEV and 7.4 months (95% CI, 4.6–11.0 months) in those treated with first-line chemotherapy with anti-EGFR antibodies (HR, 1.17; 95% CI, 0.64–2.12; *P* = .60) (Fig. 3A); median OS was 19.8 months (95% CI, 10.4–22.4 months) and 17.5 months (95% CI, 11.5–26.1 months), respectively (HR, 0.96; 95% CI, 0.52–1.78; *P* = .91) (Fig. 3B).

### Safety

Safety was assessed in 58 patients. The incidence of treatment-related adverse events is shown in Table 3. Frequencies of Grade ≥ 3 hematologic, non-hematologic, and RAM-associated adverse events were 43%, 24%, and 12%, respectively. The most frequent Grade ≥ 3 hematologic adverse event was neutropenia (40%). In addition, four (6.9%) patients experienced Grade 3 febrile neutropenia. Frequent Grade ≥ 3 non-hematologic adverse events were anorexia (10%) and diarrhea (8.6%). Frequent Grade ≥ 3 RAM-associated adverse events were hypertension (6.9%) and proteinuria (3.4%). There were no unexpected serious adverse events or treatment-related deaths.

**Table 3:** Adverse events in the safety population (*N* = 58) (*N*, total number of patients; *n*, number of patients)

	All grades		Grade ≥ 3	
	*n*	%	*n*	%
Hematological toxicity	36	62	25	43
Leukopenia	13	22	2	3.4
Neutropenia	30	52	23	40
Febrile neutropenia	4	6.9	4	6.9
Anemia	13	22	1	1.7
Thrombocytopenia	18	31	0	0
Nonhematological toxicity	51	88	14	24
Fatigue	34	59	4	6.9
Anorexia	34	59	6	10
Diarrhea	31	53	5	8.6
Nausea	30	52	1	1.7
Oral mucositis	24	41	2	3.4
Alopecia	25	43	0	0
RAM-associated toxicity	36	62	7	12
Hypertension	32	55	4	6.8
Proteinuria	9	16	2	3.4
Gastrointestinal bleeding	3	5.2	1	1.7
Thromboembolism	1	1.7	1	1.7

## Discussion

This Phase II trial was conducted to evaluate the efficacy and safety of FOLFIRI with low-dose irinotecan (150 mg/m^2^, standard dose in Japan) plus RAM as second-line treatment for mCRC in Japanese patients. The median PFS (primary endpoint) of 5.9 months (95% CI, 4.8–6.9 months) was considered acceptable according to our hypothesis that assumed the threshold and expected PFS of 4.3 months and 5.7 months, respectively. Moreover, the median OS (secondary endpoint) of 17.5 months (95% CI, 12.3–21.7 months) was comparable to those reported by previous Phase III trials, including the RAISE trial, for second-line angiogenic therapies.

The RDIs of cytotoxic agents have been shown to have significant effects on tumor response and survival benefits in patients with various cancer types, including lymphoma, ovarian cancer, lung cancer, and breast cancer [11–14]. Nakayama *et al.* reported that maintaining a high RDI of irinotecan in FOLFIRI and OX in folinic acid, 5-fluorouracil, and oxaliplatin (FOLFOX) was necessary to achieve tumor response and survival benefits in patients with mCRC. A higher RDI of irinotecan was associated with significant improvements in RR, DCR, PFS, and OS [15]. In the Japanese population of the RAISE trial, median RDIs of RAM and irinotecan were 82.9% and 63.8%, respectively [10]. In the present study, the RDI of RAM was ≥ 80% and that of irinotecan was 73.8%, which was higher than that observed in the Japanese population of the RAISE trial. Although previous reports suggested that maintaining a high RDI contributes to better outcomes, our findings indicate that setting a lower irinotecan dose (150 mg/m^2^) allowed patients to maintain a sufficient RDI and achieve comparable efficacy with reduced toxicity. This suggests that optimizing the irinotecan dose for Japanese patients may be more important than simply maintaining RDI itself.

Evidence is insufficient with regard to second-line treatment after first-line treatment with anti-EGFR antibodies, and no Phase III clinical trials have evaluated combinations of molecular-targeted agents in second-line treatment. Suzuki *et al.* compared the efficacy and safety of FOLFIRI plus RAM as second-line treatment for unresectable mCRC in patients who received first-line treatment with or without BEV. The PFS and DCR were better in BEV-naive patients than in those who had received first-line treatment with BEV [16]. In the present study, molecular-targeted agents used in first-line treatment were BEV in 44 patients (72%) and anti-EGFR antibodies in 17 patients (28%) in the ITT population; median PFS was 5.7 months (95% CI, 4.4–6.8 months) in the former group and 7.4 months (95% CI, 4.6–11.0 months) in the latter group. These findings suggest a trend toward greater efficacy in patients who did not use BEV as first-line treatment. The JACCRO CC-16 study also investigated the efficacy and safety of FOLFIRI plus RAM as second-line treatment in patients with RAS wild-type mCRC who were refractory or intolerant to OX and fluorouracil (doublet) or to fluorouracil maintenance therapy following OX, irinotecan, and fluorouracil (triplet) plus anti-EGFR antibodies as first-line therapy [17]. In that study, median PFS was 7.4 months (95% CI, 5.7–9.0) in patients who received the doublet plus anti-EGFR antibody regimen, which was comparable to our subgroup treated with anti-EGFR antibody in first-line therapy (median PFS, 7.4 months; 95% CI, 4.6–11.0). These results suggest that FOLFIRI plus RAM is effective as second-line treatment after first-line treatment with anti-EGFR antibodies.

In terms of safety, the most frequent adverse events were neutropenia and RAM-associated events, such as hypertension and proteinuria. Among Grade ≥ 3 adverse events, neutropenia, hypertension, and proteinuria were observed at frequencies of 40%, 6.8%, and 3.4%, respectively. In the Japanese population of the RAISE trial, frequencies of Grade ≥ 3 neutropenia, hypertension, and proteinuria were 59.5%, 17.6%, and 8.1%, respectively [10]. In a Phase 1b study conducted prior to the RAISE study, severe neutropenia of Grade 3 or higher was observed in five of six patients (83.3%) with 180 mg/m^2^ irinotecan [18]. The low dose of irinotecan (150 mg/m^2^) in this study resulted in lower frequency of Grade ≥ 3 neutropenia than that of Japanese population in the RAISE trial. In Japanese patients, low-dose irinotecan (150 mg/m^2^) is appropriate from safety perspectives, and FOLFIRI plus RAM therapy is performed with appropriate dose reduction and treatment delay. The incidence of hypertension was higher compared with Japanese population of the RAISE study for all grades. This may be because, in the present study, roughly 30% of patients were BEV-naive in first-line treatment, and some appeared to have developed hypertension after the use of RAM in second-line treatment. The incidence of Grade ≥ 3 hypertension and that of proteinuria were both lower than those in Japanese population of the RAISE study. All physicians performed quantitative urine tests and closely monitored blood pressure, and control of Grade ≥ 3 adverse events could be achieved through appropriate RAM dose modification and use of antihypertensive drugs when needed.

This study has several limitations. First, the single-arm design and relatively small sample size necessitate confirmation of our results in a larger cohort. Second, tumor size and response according to the RECIST criteria were not centrally evaluated. Third, the background of the present study population differed from that of the RAISE study population. All patients in the RAISE study were previously treated with BEV, whereas the present study used a heterogeneous treatment population including not only patients who received BEV but also those who received anti-EGFR antibodies as first-line treatment. Therefore, data are not directly comparable between the present study and the RAISE study.

In conclusion, we demonstrated that FOLFIRI plus RAM therapy performed in the setting of irinotecan 150 mg/m^2^ can be safely administered without decreasing the therapeutic effect. FOLFIRI with low-dose irinotecan (150 mg/m^2^, standard dose in Japan) plus RAM is a feasible second-line treatment in Japanese patients with mCRC.
